# Prevalence and factors of surgical site infections in surgical wards, Windhoek, Namibia

**DOI:** 10.4102/hsag.v30i0.2930

**Published:** 2025-07-31

**Authors:** Anna N. Haifete, Petra Brysiewicz

**Affiliations:** 1 School of Nursing and Public Health, College of Health Sciences, University of KwaZulu-Natal, Durban, South Africa; 2 School of Nursing and Public Health, Faculty of Health Sciences and Veterinary Medicine, University of Namibia, Windhoek, Namibia

**Keywords:** prevalence, surgical site infection, surgical in-patients, surgical wards, Namibia

## Abstract

**Background:**

Surgical site infection (SSI) is a prevalent healthcare-associated infection worldwide, defined as any incisional infection occurring within 30 days post-operation or within 1 year in the presence of an implant. Healthcare-associated infections represent a significant threat to patient health and continue to pose a major global challenge.

**Aim:**

This study aims to determine the prevalence of SSI and identify associated factors among patients in surgical wards who have undergone surgical procedures in Windhoek, Namibia.

**Setting:**

This study was conducted in two state hospitals in Windhoek, Namibia.

**Methods:**

This study employed a quantitative, hospital-based cross-sectional design, utilising a retrospective chart review of patients who underwent surgical procedures between March 2019 and February 2021.

**Results:**

The overall prevalence of SSI was 10.1% (95% confidence interval: 8.4% – 11.8%). Hospitals 1 and 2 had a prevalence of 11.5% and 8.7%, respectively. Factors associated with SSIs were found to be age groups 31–60 years, male patients, postoperative hospital stays ≥ 5 days, emergency surgery, abdominal and lower extremity surgeries, operation referred from other hospitals, previous history of surgery, 1 h–2 h duration of surgery and deceased patients. Diabetes mellitus, HIV/AIDS, cancer, malignancy and multiple comorbidities were also noted to increase the risk of SSIs.

**Conclusion:**

The prevalence of SSI in this study remains high, and therefore, context-based interventions should focus on the factors identified to guide the effective management of patients.

**Contribution:**

This study provides evidence to improve surgical site infections in Namibia.

## Introduction

### Background of the study

Surgical site infections (SSIs) represent a prevalent category of healthcare-associated infections (HAIs) (World Health Organization [WHO] 2018). Healthcare-associated infections pose a significant threat to patient health and continue to represent a major challenge for health care providers worldwide (Mengistu et al. 2023). These SSIs refer to infections that occur 30 days after an operation or within 1 year after implantation (U.S. Centers for Disease Control and Prevention 2024). This condition continues to be a significant contributor to global morbidity and mortality. Therefore, it is important to investigate SSI. The United Nations Sustainable Development Goal (SDGs) 3 aims to ensure healthy lives and promote well-being across all nations (United Nations 2023). The SDG target 3.8 is to achieve Universal Health Coverage (UHC), of which SSIs are a key indicator of health care quality and safety (World Health Organization & the World Bank 2023). To address this, the WHO (2024) has developed a comprehensive set of strategies, including infection prevention and control, in-service education and training curricula to help nations address SSIs and other HAIs.

A prevalence survey conducted in Serbia by Suljagic et al. (2021) discovered higher HAIs among surgical patients (261/3626 or 7.2%) than those among non-surgical patients (258/8852 or 2.9%). Ansari et al. (2019) in Pakistan highlighted that SSIs are more common in older patients above 50 years (11.4%). And diabetes mellitus is a risk factor associated with SSI (Ansari et al. 2019; Sattar et al. 2019). Further, pre-existing infection, malnutrition, obesity, low serum albumin, elderly and smoking are patient-related factors associated with SSI (Ansari et al. 2019). In Canada, Alkaaki et al. (2019) reported the likelihood of emergency surgeries developing SSI rather than elective surgery. In China, Hou et al. (2020) discovered that preoperative hospital stays and contaminated or dirty wounds were significantly associated with an increasing risk of SSI. In Brazil, De Carvalho et al. (2017) identified the length of preoperative hospital stays of more than 24 h, extended duration in hours of surgery, clean-contaminated, contaminated and dirty wounds, and American Society of Anaesthesiologists Physical Status Score (ASA index) as risk factors. A study by Gurunthalingam et al. (2023) in India recorded that only 10 (2.53%) out of 394 case records received a suitable antibiotic. The length of surgical antibiotic prophylaxis (SAP) was correct only in 24 (6.53%) case records, and the time of SAP administration was correct in 204 (50.76%) case records.

In low- and middle-income countries (LMICs), SSIs account for up to 60% of HAIs (Sefah et al. 2022). A large-scale adult study in Africa by Biccard et al. (2018) indicated 1156 (10.2%) postoperative infection complications out of 10 970 patients, of whom 112 (9.7%) died. Prevalence of SSI was reported as 7.81% in Benin (Degbey et al. 2021) and patients who were > 60 years of age were at great risk of contracting it. Studies in Ethiopia by Alamrew et al. (2019) and Awoke, Arba and Girma (2019) reported the likelihood of emergency surgeries developing SSI rather than elective surgery. Gedefaw et al. (2018) in Ethiopia also reported diabetes mellitus as a risk factor associated with SSI. In Ghana, compliance with SAP because of the appropriate choice of antimicrobials was 400 (67.0%), and the duration of SAP was appropriate only in 52 (8.7%) (Sefah et al. 2022).

The socio-economic burden imposed by the costs of SSI remains a significant problem for many countries (Maraş & Sürme 2023). In the United States, re-admission for one patient with SSI costs more than $159 000 (Fourier 2020). In India, Monahan et al. (2020) discovered that the additional medical cost of SSI in the LMICs ranges between $174 000 and $29 610, and in high-income European countries, it ranges from $21 000 to $34 000. In colorectal surgery, additional medical costs stood at $18 101. This led to 22.72 extra days in the hospital. A study conducted in various LMICs in Europe by Costabella et al. (2023) highlighted that inadequate surgical infrastructure, human resources and equipment contribute to high SSI costs. Namibia is no exception when it comes to HAIs, including SSIs (Ministry of Health and Social Services [MoHSS] 2023). To address this, the MoHSS of Namibia developed the Namibian Infection Prevention and Control Action Plan 2023/2024-2026/2027, and one of the strategic objectives is to develop and implement evidence-based guidelines to reduce HAIs. However, the average SSI cost for patients and additional costs were not reported for Namibia.

### Aim

This study aims to determine the prevalence of SSI and identify the factors associated with them among surgical ward patients who underwent a surgical procedure in Windhoek, Namibia.

## Research methods and design

### Study design

This study employed a quantitative, hospital-based cross-sectional design, utilising a retrospective chart review of patients who underwent surgical procedures from March 2019 to February 2021.

### Research setting

This study took place in Namibia at two state training hospitals that serve as the intermediate referral hospital (Hospital 1) and the main national referral hospital (Hospital 2). Hospital 1, with a bed capacity of 843, has the following surgical areas: two adult surgical, one paediatric surgical, two orthopaedic, one gynaecology ward and a theatre. Hospital 2 is a training hospital with an 850-bed capacity. This hospital consists of various wards or departments, among them: two adult surgical, one paediatric surgical, two adult urology or gynaecology, one intensive care unit (ICU), one paediatric ICU, one surgical and trauma ICU, one cardiothoracic surgery ward and one theatre. This hospital also has a private ward for all disciplines where private patients who underwent surgery are admitted.

### Population, sample size and sampling method

The population for these wards was similar both pre-COVID-19 and during COVID-19, ranging between 4880 and 4908 per annual reporting period at both hospitals. Based on the advice of a statistician, the study estimated the likelihood of a risk factor in terms of odds ratios, of which 1.5 is considered small and desirable, while 3.5 and 9 are considered medium and large, respectively. Using G Power for sample size calculation, it was estimated that a minimum sample size of 1246 records was required to detect effect sizes of at least 2.9 about 80% of the time (power of test). A sample size of 1248 patients’ records was divided equally into 4 time periods, 2 periods for each hospital (1248 divided by 4 periods = 312); thus, a systematic sampling method for March 2019 to February 2020 and March 2020 to February 2021 patients’ charts was used for each hospital.

#### Inclusion criteria

- All charts for patients who underwent surgery during the study period from March 2019 to February 2021 were included.

#### Exclusion criteria

- Patients who stayed in the hospital for less than 24 h postoperatively were excluded.
- Ophthalmic surgery during the same period was excluded.

### Research tool and data collection procedure

Data were collected using a checklist developed by the researchers and based on the study by Awoke et al. (2019). The tool was subdivided into four sections: demographic information, surgery-related factors, comorbidities, wound-related factors, and anaesthesia and medication-related factors (see Box 1 for additional details).

BOX 1 Explanation of data collection tool.

**Table:** 

**Demographic factors:** Age of patient Gender Surgical area admitted to Admission period (how long in hospital) Operation date Patient’s place of residence	**Comorbidities and wound-related factors:** Duration of surgery Amount of blood loss during surgery Implant insertion done Installation of drain American Society of Anesthesiologists Physical Status Score Associated chronic comorbidity Wound care ordered Frequency of wound care Wound care was chartered as ordered
**Surgery-related factors:** Preoperative hospital stays Postoperative hospital stays Type of surgery Wound classification (e.g. dirty, clean-contaminated, contaminated and clean) Body region where operation was done Other institutions where operation took place Previous history of surgery Patient mode of discharged (e.g. discharged home or diseased)	**Anaesthesia and medication-related factors:** Type of anaesthesia Duration of anaesthesia Antibiotic prophylaxis

The SSI was diagnosed and confirmed by a registered medical practitioner within 30 days following surgery or up to 1 year in case of an implant. Wound infection was identified based on the presence of local inflammatory changes, including oedema, redness, warmth and pus discharge.

The study ensured the content and face validity of the data collection tool as it was guided by the tool used by Awoke et al. (2019), data routinely captured in the hospital records and verified by a statistician. The reliability of the study was incorporated in the sample size calculations, where a minimum sample was determined to detect the appropriate effect size and reduce Type I and Type II errors.

### Data collection process

The data collection process (February 2023 to July 2023) started after ethics approval by the University of KwaZulu-Natal and permission from the Ministry of Health and Social Services in Namibia and medical superintendents of the two hospitals. The researcher (ANH) drew every 4th patient’s chart in health records based on the systematic sampling method. Data collection was a lengthy process, with each patient chart taking approximately 30 min–40 min to complete.

### Data analysis

Data were cleaned and analysed, with the help of a statistician, using IBM Statistical Package for the Social Sciences (IBM SPSS) version 29. The data were coded before being entered into IBM SPSS version 29. Descriptive statistics, such as percentages and frequencies, were used to analyse nominal and ordinal data. The frequency of each response was expressed as a percentage. The prevalence of SSIs was computed as a percentage. Chi-square tests were performed to determine whether there were associations between independent variables such as age and gender and the dependent variable (SSI). Binary logistic regression was computed to determine the extent of the associations between the independent variables and the dependent variable (SSI); 95% confidence intervals were used to determine the statistical significance of the odds ratios, and *p*-values of less than 0.05 were used to determine the statistical significance of the results of the Chi-square tests.

### Ethical considerations

Ethical clearance was received from the University of KwaZulu-Natal’s Biomedical Research Ethics Committee (BREC) on 11 September 2022 (clearance no: BREC/00004372/2022) and denotes compliance with South African National Research Ethics Guidelines (2015), and permission to conduct the study was from the Ministry of Health and Social Services of Namibia (MoHSS) Research Committee.

## Results

All selected files were traced at both sites. However, the following variables had missing data: the amount of blood loss (77%), wound classification (86%), American Society of Anesthesiologists Physical Status Score (23%), order for wound care (17%), orders showing how often to clean the wound (59%), wound care charted as ordered (49%), duration of anaesthesia (6%) and antibiotic prophylaxis (24%). All missing data are reported in the results.

### Prevalence of surgical site infections

The overall prevalence of SSIs was 10.1% (95% confidence interval: 8.4% – 11.8%). Hospitals 1 and 2 had a prevalence of 11.5% (*n* = 72) and 8.7% (*n* = 54), respectively.

### Demographic factors

Chi-square tests revealed that there was an association between age and SSIs and between gender and SSIs (*p* < 0.05). However, no associations were noted between COVID-19 period, residence and type of ward with SSIs (*p* > 0.05). Age category 31–60 years had a statistically significantly lower likelihood of SSIs than age category < 1–30, crude odds ratio (COR) = 0.38 (95% CI [confidence interval]: 0.25–0.58). This means that patients aged 31–60 years had a 62% lower chance of developing SSIs than those in the age category < 1–30. Male patients were 1.69 times more likely to develop SSIs compared to female patients, COR = 1.69 (95% CI: 1.16–2.47). This study shows more SSIs in patients operated during pre-COVID-19 (March 2019 to February 2020) than during COVID-19 (March 2020 to February 2021). However, the COR for SSIs during COVID-19 compared to the pre-COVID-19 period was 1.38 (95% CI: 0.95–2.00), indicating that there was no significant difference in the likelihood of developing the infections between those admitted before COVID-19 (11.6%) and during the COVID-19 pandemic (8.6%) (*p* = 0.053). More details are in Table 1.

**TABLE 1:** Demographic factors (*N* = 1248).

Variables	Category	SSI				Crude Odds Ratio	95% CI	Chi-square test *p*-value
		Yes		No				
		*n*	%	*n*	%			
Age groups (years)	< 1–30	32	5.9	508	94.1	1	-	*p*< 0.001 *
	31–60	79	14.3	475	85.7	**0.38**	**0.25–0.58**	
	> 60	15	9.7	139	90.3	0.58	0.31, 1.11	
Gender	Male	77	12.5	540	87.5	**1.69**	**1.16–2.47**	*p*< 0.006 *
	Female	49	7.9	582	92.2	1	-	
COVID-19	Pre COVID-19	72	11.6	551	88.4	1.38	0.95–2.00	*p* = 0.053
	During COVID-19	54	8.6	571	91.4	1	-	
Residence	Urban	88	9.7	823	90.3	0.86	0.56–1.32	*p* = 0.485
	Rural	32	11.1	257	88.9	1	-	
	NS	6	12.5	42	87.5	1.15	0.45–2.91	
Type of ward	Private	3	5.3	54	94.7	0.48	0.15–1.57	*p* = 0.216
	Public	116	10.3	1008	89.7	1	-	
	NS	7	10.4	60	89.6	1.01	0.45–2.27	

Note: Bold numbers are statistically significant.

COR, Crude odds ratio; NS, not stated; SSI, surgical site infections; CI, confidence interval.

* , Statistically significant at *p* < 0.05.

### Surgery-related factors

Chi-square tests revealed that postoperative duration of hospital stay, the type of surgery done, wound classification, previous history of surgery, patient mode of discharge from the hospital and the operation being performed at another hospital were all statistically significantly related to the occurrence of SSIs (*p* < 0.05). Binomial logistic regression revealed that participants with more than 5 days of postoperative hospital stay were about three times more likely to develop SSIs than those who stayed less than 5 days, COR = 3.18 (95% CI: 2.13–4.75). Participants who had emergency or urgent surgery were almost three times more likely to develop SSIs than those who had elective surgery, COR = 2.71 (95% CI: 1.86–3.95). Participants who had clean-contaminated or contaminated wounds were statistically significantly more likely to develop SSIs than those with clean wounds, COR = 81.20 (95% CI: 20.90–314.97) and COR = 72.50 (95% CI: 11.21–468.77), respectively. Additionally, participants who had abdominal surgery or surgery of the lower extremities were about three times more likely to develop SSIs than those who had head and neck surgery, COR = 3.22 (95% CI: 1.15–8.97) and COR = 3.10 (95% CI: 1.01–9.50), respectively. Participants with a previous history of surgery or referred from another institution were more likely to develop SSIs, COR = 2.14 (95% CI: 1.37–3.34) and COR = 5.38 (95% CI: 2.99–9.69), respectively. Furthermore, participants who died before discharge were more than three times more likely to have SSIs than those who were discharged, COR = 3.39 (95% CI: 1.40–8.21). More details are in Table 2.

**TABLE 2:** Surgery-related factors (*N* = 1248).

Variables	Category	SSI				Crude odds ratio	95% CI	Chi-square test *p*-value
		Yes		No				
		*n*	%	*n*	%			
Preoperative duration of hospital stays (in days)	1–4	114	90.5	1012	90.2	1.03	0.55–1.93	*p* = 0.537
	≥ 5	12	9.5	110	9.8	1	-	
Postoperative duration of hospital stays (in days)	1–4	37	5.5	639	94.5	1	-	*p* < 0.001 *
	≥ 5	89	15.6	483	84.4	**3.18**	**2.13–4.75**	
Type of surgery	Elective	52	6.6	736	93.4	1	-	*p* < 0.001 *
	Emergency or urgent	74	16.1	386	83.4	**2.71**	**1.86–3.95**	
Wound classification	Clean	5	3.3	145	96.7	1	-	*p* < 0.001 *
	Clean-contaminated	14	73.7	5	26.3	**81.20**	**20.90–314.97**	
	Contaminated	5	71.4	2	28.6	**72.50**	**11.21–468.77**	
	NC	102	9.5	970	90.5	-	-	
Where surgery was done (body regions)	Head and neck	4	4.0	97	96.0	1	-	*p* = 0.061
	Thorax	12	7.5	147	92.5	1.98	0.62–6.32	
	Abdomen	86	11.7	648	88.3	**3.22**	**1.15–8.97**	
	Upper extremity	7	6.7	97	93.3	1.75	0.50–6.17	
	Lower extremity	17	11.3	133	88.7	**3.10**	**1.01–9.50**	
Operation referred from another institution	Yes	19	33.9	37	66.1	**5.38**	**2.99–9.69**	*p* < 0.001*
	No	107	9.0	1122	89.9	1	-	
Previous history of surgery	Yes	30	17.3	143	82.7	**2.14**	**1.37–3.34**	*p* < 0.001*
	No	96	8.9	979	91.1	1	-	
Duration of surgery	< 1 h	12	5.5	206	94.5	1	-	*p* = 0.057
	1–2 h	102	11.7	769	88.3	**2.28**	**1.23–4.22**	
	3–4 h	9	9.9	82	90.1	1.88	0.77–4.64	
	> 4 h	2	15.4	11	84.6	3.12	0.62–15.70	
	NS	1	1.8	54	98.2	-	-	
Amount of blood loss during surgery	< 500 mL	31	24.6	96	75.6	1	-	*p* = 0.595
	500–1500 mL	38	29.5	91	70.5	1.29	0.74–2.25	
	> 1500 mL	9	31.0	20	69.0	1.39	0.58–3.38	
	NS	48	5.0	915	95.0	-	-	
Implant inserted at site of surgery	Yes	17	11.8	126	88.1	1.20	0.70– 2.07	*p* = 0.505
	No	107	10.1	954	89.9	1	-	
	NA	2	4.5	42	95.5	-	-	
Installation of the drain	Yes	25	12.4	177	87.6	1.28	0.80–2.05	*p* = 0.293
	No	100	9.9	909	90.1	1	-	
	NA	1	2.7	36	97.3	-	-	
Patient mode of discharge from the hospital	Discharged	107	9.4	1037	90.6	1	-	
	Dead	7	25.9	20	74.1	**3.39**	**1.40–8.21**	***p* = 0.004**
	NR	12	15.6	65	84.4	-	-	

Note: Bold numbers are statistically significant.

COR, Crude odds ratio; NC, not classified; NS, not stated; NA, not applicable; NR, not recorded; SSI, surgical site infections; CI, confidence interval.

* , Statistically significant at *p* < 0.05.

### Comorbidities and wound-related factors

Chi-square tests revealed that ASA index, associated chronic comorbidities, if wound care was ordered and frequency of wound care ordered were statistically significantly associated with SSIs (*p* < 0.05). However, charting of wound care as ordered was not statistically significantly associated with the occurrence of SSIs (*p* > 0.05). In binomial logistic regression, participants with ASA III/ASA IV were about 4 times more likely to develop SSIs than those with ASA I/ASA II, COR = 4.25 (95% CI: 2.38–7.60). Compared with participants without comorbidities, participants with diabetes mellitus, COR = 9.87 (95% CI: 5.78–16.84); HIV/AIDS, COR = 3.40 (95% CI: 1.84–6.27); cancer, COR = 9.28 (95% CI: 3.50–24.66); or multiple comorbidities, COR = 3.14 (95% CI: 1.65–5.97) were statistically significantly more likely to develop SSIs. More details are in Table 3.

**TABLE 3:** Comorbidities and wound-related factors (*N* = 1248).

Variables	Category	SSI				Crude odds ratio	95% CI	Chi-square test *p*-value
		Yes		No				
		*n*	%	*n*	%			
ASA index	ASA I/ASA II	73	8.2	817	91.8	1	-	*p* < 0.001*
	ASA III/ASA IV	19	27.5	50	72.5	**4.25**	**2.38–7.60**	
	NS	34	11.8	255	88.2	-	-	
Associated chronic comorbidity	Diabetes mellitus	31	38.3	50	61.7	**9.87**	**5.78–16.84**	*p* < 0.001*
	Hypertension	6	4.5	128	95.5	0.75	0.31–1.78	
	HIV/AIDS	16	17.6	75	82.4	**3.40**	**1.84–6.27**	
	Cancer or malignancy	7	36.8	12	63.2	**9.28**	**3.50–24.66**	
	Multiple comorbidities	14	16.5	71	83.5	**3.14**	**1.65–5.97**	
	Others	4	15.4	22	84.6	2.89	0.96–8.73	
	None	48	5.9	764	94.1	1	-	
If wound care was ordered	Yes	102	18.0	466	82.0	1	-	*p* < 0.001*
	No	15	3.5	412	96.5	**0.17**	**0.10–0.29**	
	NS	9	3.6	244	96.4	-	-	
Frequency of wound care ordered	1 × day	47	16.0	247	84.0	1	-	*p* < 0.001*
	2 × day	33	26.4	92	73.6	**1.89**	**1.14–3.12**	
	Three times a day	5	10.9	41	89.1	0.64	0.24–1.71	
	Alternative days	6	14.6	35	85.4	0.90	0.36–2.26	
	Continuous vacuum dressing	4	100	0	0.0	-	-	
	NS	31	4.2	707	95.8	-	-	
Wound care charted as ordered	Yes	35	15.2	196	84.8	1	-	*p* = 0.554
	No	69	17.0	338	83.0	1.14	0.73–1.78	
	NS	22	3.9	542	97.0	-	-	
	NA	0	0.0	46	100	-	-	

Note: Bold numbers are statistically significant.

ASA, American Society of Anaesthesiologists Physical Status Score; COR, Crude odds ratio; NS, not stated; NA, not applicable; SSI, surgical site infections; CI, confidence interval; HIV, Human Immunodeficiency Virus; AIDS, Acquired Immunodeficiency Syndrome.

* , Statistically significant at *p* < 0.05.

### Anaesthesia and medication-related factors

Chi-square tests revealed that the duration of anaesthesia and the use of prophylactic antibiotics were statistically significantly associated with SSIs (*p* < 0.05). However, the type of anaesthesia given was not statistically significantly associated with the occurrence of SSIs (*p* > 0.05). In binomial logistic regression, participants who received spinal anaesthesia were 78% less likely to develop SSIs than those who received regional or local anaesthesia, COR = 0.22 (95% CI: 0.06–0.80). Participants who had anaesthesia for more than 90 min were statistically significantly more likely to develop SSIs than those who had anaesthesia for less than 30 min, COR = 3.71 (95% CI: 1.71–8.81). Furthermore, participants who did not receive prophylactic antibiotics were statistically significantly less likely to develop SSIs, COR = 0.18 (95% CI: 0.08–0.39). More details are in Table 4.

**TABLE 4:** Anaesthesia and medication-related factors (*N* = 1248).

Variables	Category	SSI				Crude odds ratio	95% CI	Chi-square test *p*-value
		Yes		No				
		*n*	%	*n*	%			
Type of anaesthesia given	General	112	10.4	968	89.6	0.53	0.23–1.23	*p* = 0.062
	Spinal	4	4.6	83	95.4	**0.22**	**0.06–0.80**	
	Regional or local	7	2.6	32	97.4	1	-	
	NS	3	7.5	37	92.5	0.37	0.09–1.55	
Duration of anaesthesia given	< 30 min	8	6.5	116	93.5	1	-	*p* < 0.001*
	30 min–60 min	20	6.1	307	93.9	0.94	0.40–2.20	
	61 min–90 min	54	10.0	487	90.0	1.61	0.74–3.47	
	> 90 min	37	20.4	144	79.6	**3.71**	**1.71–8.81**	
	NS	7	9.3	68	90.7	1.49	0.52–4.30	
Antibiotic prophylaxis given	Yes	105	14.6	613	85.4	1	-	*p* < 0.001*
	No	7	3.0	229	97.0	**0.18**	**0.08–0.39**	
	NS	14	4.8	280	95.2	-	-	

Note: Bold numbers are statistically significant.

COR, Crude odds ratio; NS, not stated; SSI, surgical site infections; CI, confidence interval.

* , Statistically significant at *p* < 0.05.

## Discussion

The study addressed the prevalence of SSIs and its associated factors among postoperative patients. In this study, the overall prevalence of SSIs was 10.1%, which is higher than that in a study by Mohan et al. (2023) (5.6%) from India, Degbey et al. (2021) from Benin (7.8%) and Gedefaw et al. (2018) from Ethiopia (9.4%). However, this study’s prevalence is lower than that reported in a study by Mezemir et al. (2020) from Ethiopia (24.6%). It is important to note that this study was not able to follow up post-discharge and some patients may have developed SSI in the community. The higher prevalence of SSIs in this study might be attributed to limited health care infrastructure and fragile health system contributing to inefficient infection control practices in the hospitals, inadequate environmental hygiene and a high number of people using the same theatre (Mezemir et al. 2020).

This study shows a higher percentage of SSIs in patients operated pre-COVID-19 (11.6%) compared to those operated during COVID-19 (8.6%), but it was not statistically significant. This is similar to a study in India by Pantvaidya et al. (2022) which showed a 4.2% decrease in SSIs during the COVID-19 pandemic, which was statistically significant, and a study in Germany by Chacon-Quesada, Rohde and Von der Brelie (2021) which indicated a 2.9% pre-COVID-19 and a 1.4% during COVID-19, which was also statistically significant. These findings may be attributed to the reduced number of theatre cases and improved infection prevention and control measures implemented during the COVID-19 period.

Participants in the age group 31–60 years had a lower likelihood of developing SSIs than those in the less than 30 years age group. This is contrary to the results of studies by Mohan et al. (2023) and Shakir et al. (2021), which reported that older age groups have a higher likelihood of developing SSIs. Older age groups are expected to have an increased risk of developing SSIs because of a decline in their immune system’s ability to fight infections and an increase in the occurrence of comorbidities (Amruthan, Reddy & Pyadala 2017). Male patients were 1.69 times more likely to develop SSIs compared to female patients. This was supported by a study by Lakoh et al. (2022). The heightened probability may be linked to the characteristics of the infected wounds presented by male patients in the surgical department, as they generally exhibit greater severity and contamination (Mohan et al. 2023).

Participants who spent ≥ 5 days in the hospital postoperatively were more likely to develop SSIs than those who spent fewer days. Mezemir et al. (2020) also reported a higher likelihood in patients who spent > 14 days. This finding may be attributable to contamination of incisions by nosocomial bacteria that may be resistant to several antibiotics. Patients with emergency/urgent cases were 0.37 more likely to develop SSIs compared to elective cases. This higher likelihood of SSIs in emergency surgeries may be because of the very urgent preparation of patients that may lead to dirtier and contaminated wounds (Papadopoulos et al. 2021).

According to the studies by Mohan et al. (2023), Awoke et al. (2019) and Mezemir et al. (2020), the likelihood of SSIs is higher with abdominal body region surgery than with the head and neck region. Abdominal surgeries are more prone to bacterial contamination and, therefore, more likely to develop SSIs than other regional surgeries (Jahangir et al. 2024).

Operations referred from other health facilities were 5.21 times more likely to develop SSIs. This may be attributed to inadequate infection prevention and control measures in referring facilities, which are often underresourced (Lowe et al. 2021). Previous history of surgery was associated with a higher likelihood of SSIs, and this might be because of prior exposure to resistant microorganisms, as reported by Awoke et al. (2019). In this study, patients who died before discharge were more likely to have SSIs than those who were discharged. Similar findings were reported in a study by Costabella et al. (2023), which revealed that SSIs contributed to 38% of postoperative deaths. This is consistent with another study by Nepogodiev et al. (2019), which confirmed postoperative mortality being the third highest contributor to global deaths, with SSIs playing a significant role.

Participants with diabetic mellitus had a higher likelihood of developing SSIs than those with no comorbidities. This concurs with the findings of studies by Shakir et al. (2021) and Papadopoulos et al. (2021). This could be attributed to poor peripheral oxygen supply because of altered inflammatory response and metabolic imbalances after surgery (Awoke et al. 2019; He et al. 2023). Moreover, these patients usually have an inferior nutritional status (Grzywacz, Lubas & Niemczyk 2023) and possibly more comorbidities (Awoke et al. 2019). Malignancy, HIV/AIDS and having multiple comorbidities were also reported to be associated with a higher risk of developing SSIs. Liu et al. (2023) supported the argument that HIV/AIDS patients with lower CD counts have a higher risk of developing SSI.

This study revealed that participants who had spinal anaesthesia had a lower likelihood of developing SSIs than those who had regional or local anaesthesia. This study revealed that participants who had surgery for 1 h–2 h were about two times more likely to develop SSIs. Similar findings were reported in a systematic review by Cheng et al. (2017). These findings are possibly because of an increase in open incisions in the environment, thus increasing the risk of bacterial contamination (Shiferaw et al. 2020). In this study, 4.8% of participants who developed SSIs had no antibiotic prophylaxis use recorded as per standard guidelines. This aligns with the findings of Sefah et al. (2022), who reported that adherence to the Ghana Standard Treatment Guidelines (STGs) was just 2.5%. In comparison, adherence of surgical cases to the guidelines set by the American Society of Health-System Pharmacists (ASHP) and Indian Council of Medical Research (ICMR) was below 1% (Gurunthalingam et al. 2023). This can lead to some emerging infections and antibiotic resistance, increasing health care costs (Tiri et al. 2020).

### Limitations

Additional data to differentiate superficial, deep and organ SSIs, as well as post-discharge surveillance, would add to the study findings. Developing SSIs after discharge could not be detected by the researcher, thus estimating the true burden of the infections.

The large amount of missing data led to the skewness of the findings. The data collected during the COVID-19 pandemic are inconsistently documented as overwhelmed nurses may not have had enough time to document all patients’ details.

### Recommendations

The study recommends that evidence-based preventive practices should be integrated into preoperative and postoperative periods to prevent SSIs. Further study on the prevalence of surgical SSIs should be conducted at the peripheral health facilities where patients are referred after discharge. A longitudinal study with a thorough methodology needs to be conducted to identify more risk factors. Educational interventions to address data missingness reporting as a result of incomplete recording are required. Hospitals must improve their filing systems of patient records.

## Conclusion

The study revealed a higher prevalence of SSIs in Namibia than that in high-income settings. The factors associated with SSIs are age groups 31–60 years, male patients, postoperative hospital stays ≥ 5 days, emergency surgery, abdominal region and lower extremity surgeries, operation referred from other hospitals, previous history of surgery, 1 h–2 h duration of surgery and deceased patients. Also, diabetes mellitus, HIV/AIDS, malignancy and multiple comorbidities were risk factors for SSIs.
